# Impact of Familial Loading on Prefrontal Activation in Major Psychiatric Disorders: A Near-Infrared Spectroscopy (NIRS) Study

**DOI:** 10.1038/srep44268

**Published:** 2017-03-15

**Authors:** Kazutaka Ohi, Takamitsu Shimada, Hiroaki Kihara, Toshiki Yasuyama, Kazuyuki Sawai, Yukihisa Matsuda, Kazuaki Oshima, Hiroaki Okubo, Yusuke Nitta, Takashi Uehara, Yasuhiro Kawasaki

**Affiliations:** 1 Department of Neuropsychiatry, Kanazawa Medical University, 1-1 Daigaku, Uchinada, Ishikawa, 920-0293, Japan; 2 Project Research Center, Kanazawa Medical University, 1-1 Daigaku, Uchinada, Ishikawa, 920-0293, Japan

## Abstract

Family history (FH) is predictive of the development of major psychiatric disorders (PSY). Familial psychiatric disorders are largely a consequence of genetic factors and typically exhibit more severe impairments. Decreased prefrontal activity during verbal fluency testing (VFT) may constitute an intermediate phenotype for PSY. We investigated whether familial PSY were associated with a greater severity of prefrontal dysfunction in accordance with genetic loading. We measured prefrontal activity during VFT using near-infrared spectroscopy (NIRS) in patients with schizophrenia (SCZ, *n* = 45), major depressive disorder (MDD, *n* = 26) or bipolar disorder (BIP, *n* = 22) and healthy controls (HC, *n* = 51). We compared prefrontal activity among patients with or without FH and HC. Patients in the SCZ, MDD and BIP patient groups had lower prefrontal activity than HC subjects. Patients with and without FH in all diagnostic groups had lower prefrontal activity than HC subjects. Moreover, SCZ patients with FH had lower prefrontal activity than SCZ patients without FH. When we included patients with SCZ, MDD or BIP in the group of patients with PSY, the effects of psychiatric FH on prefrontal activity were enhanced. These findings demonstrate the association of substantially more severe prefrontal dysfunction with higher genetic loading in major psychiatric disorders.

Schizophrenia, major depressive disorder and bipolar disorder are common and complex major psychiatric disorders. The lifetime risks of schizophrenia, major depressive disorder and bipolar disorder are approximately 0.5%, 12% and 1%, respectively^1^,^2^,^3^. These disorders are characterized by clinical and genetic heterogeneity that manifests in a broad range of deficits. Twin studies have indicated high degrees of heritability for schizophrenia (approximately 80%)^4^, major depressive disorder (45%)^5^,^6^ and bipolar disorder (85%)^7^,^8^. Psychiatric family history is a major risk factor for these disorders^9^,^10^. Patients who present with major psychiatric disorder often have a history of familial loading^11^. People with first- or second-degree relatives who have a family history of schizophrenia, major depressive disorder or bipolar disorder have 10-, 2- and 8-fold higher risks of developing schizophrenia, major depressive disorder or bipolar disorder, respectively, compared with the general population^10^,^12^,^13^,^14^. Premorbid neuropsychological deficits are found in a substantial proportion of children who later develop schizophrenia, especially in the subgroup of schizophrenia patients with a family history^15^. Moreover, there is a substantial body of literature describing associations between a family history of schizophrenia and an impaired intelligence quotient (IQ), attention and memory^16^,^17^,^18^, decreased brain volumes^19^ and brain dysfunction^20^ in otherwise healthy children, adolescents and adults. The Consortium on the Genetics of Schizophrenia (COGS) Family Study has evaluated neurocognitive and neurophysiological intermediate phenotypes in schizophrenia probands and their families and has found greater heritability of intermediate phenotypes among schizophrenia families with greater genetic vulnerability to illness^21^. Furthermore, polygenic risk score (PGRS) analysis based on a genome-wide association study (GWAS) has indicated that the aggregate effect of genome-wide genetic variants is significantly greater for schizophrenia patients with a family history than for schizophrenia patients without a family history^22^. These findings suggest that familial clustering is largely due to genetic factors and may therefore highlight the potential utility of family history as a predictive tool for preventing major psychiatric disorders^23^. The utility of family history in preventive interventions has been demonstrated in common diseases, such as diabetes, cardiovascular disease and some types of cancer^23^.

Imaging genetics represent a powerful approach for exploring the relationship between brain function and genetic variations that increase the risk of psychiatric disorders^24^. Near-infrared spectroscopy (NIRS) is a functional neuroimaging technology that enables non-invasive detection of cortical surface changes in oxyhemoglobin (oxy-Hb) and deoxyhemoglobin (deoxy-Hb) concentrations during task performance. The changes are assumed to reflect regional cerebral blood volumes. NIRS is suitable for clinical application, particularly in psychiatric disorders, because of its relatively low cost, easy set up and relative insensitivity to motion artifact^25^. NIRS studies using the letter version of the verbal fluency test have revealed higher prefrontal oxy-Hb levels in healthy subjects during task performance^26^. In contrast, decreased prefrontal activity during the verbal fluency test has been reported among patients with major psychiatric disorders, including schizophrenia^25^,^27^,^28^,^29^,^30^ and mood disorders^30^,^31^,^32^,^33^. Furthermore, the rise of prefrontal blood flow during task performance is delayed in patients with schizophrenia and bipolar disorder compared with patients who have major depressive disorder^34^. Moreover, twin studies of healthy adults have indicated high heritability (66–75%) of prefrontal activity changes measured by NIRS^35^. Therefore, altered prefrontal activity is a reliable intermediate phenotype for genetic imaging studies in psychiatric disorders^36^.

To the best of our knowledge, no previous study has examined the association of a psychiatric family history with prefrontal activity changes measured by NIRS in major psychiatric disorders, including schizophrenia, major depressive disorder and bipolar disorder. We hypothesized that patients with a psychiatric family history would have greater reductions in prefrontal activity than those without a family history because psychiatric family history is the strongest known risk factor for major psychiatric disorders. In the current study, we investigated the influences of psychiatric familial loading on prefrontal activity, as measured by multi-channel NIRS during the verbal fluency test in patients with schizophrenia, major depressive disorder or bipolar disorder and healthy controls.

## Methods and Materials

### Subjects

Subjects for this study consisted of 45 patients with schizophrenia (16 males/29 females, mean age ± SD: 35.4 ± 9.1 years), 26 patients with major depressive disorder (17 males/9 females, 41.1 ± 12.7 years), 22 patients with bipolar disorder (13 males/9 females, 39.9 ± 12.5 years) and 51 healthy subjects (33 males/18 females, 35.7 ± 11.9 years). All subjects were of Japanese descent, and all were biologically unrelated to at least the second degree. Patients were recruited from both the outpatient and inpatient populations at Kanazawa Medical University Hospital. Each patient with schizophrenia, major depressive disorder or bipolar disorder had been diagnosed by at least two trained psychiatrists on the basis of unstructured clinical interviews, medical records and clinical conferences. Diagnoses were made according to criteria in the fifth edition of the *Diagnostic and Statistical Manual of Mental Disorders* (DSM-5). Healthy controls were recruited through local advertisements and from among hospital staff at Kanazawa Medical University. Healthy controls were evaluated using unstructured psychiatric interviews to exclude individuals who had had current or past contact with psychiatric services or who had received psychiatric medication. Subjects were excluded from analysis if they had neurological or medical conditions that could affect the central nervous system, including atypical headaches, head trauma with loss of consciousness, chronic lung disease, kidney disease, chronic hepatic disease, active cancer, cerebrovascular disease, epilepsy, seizures, substance-related disorders or mental retardation. Demographic information is shown in Table 1. The mean age, handedness ratio and duration of illness did not significantly differ among diagnostic groups (*p* > 5.00 × 10^−2^), whereas the gender ratio, number of years of education, estimated premorbid IQ, verbal fluency testing performance, chlorpromazine equivalents used (CPZ-eq.) and age of onset differed significantly among the groups (*p* < 5.00 × 10^−2^). Current clinical symptoms in patients with schizophrenia, major depressive disorder or bipolar disorder were evaluated using the Positive and Negative Syndrome Scale (PANSS)^37^, 17-item Hamilton Rating Scale for Depression (HAMD-17)^38^ or Young Mania Rating Scale (YMRS)^39^, respectively. In the current study, we focused on the effects of a family history of psychiatric illness on prefrontal activity. Family history was determined on the basis of information from patients and family members obtained through clinical interviews and/or checklists. A positive family history (+) was defined as at least one first- or second-degree relative with the following: (i) a diagnosis of any psychiatric disorder [autism, dementia, anxiety disorders or major psychiatric disorder (schizophrenia, major depressive disorder or bipolar disorder)], (ii) a diagnosis of any major psychiatric disorder (schizophrenia, major depressive disorder or bipolar disorder) or (iii) a diagnosis in both the proband and a relative of major psychiatric disorder (schizophrenia, major depressive disorder or bipolar disorder, e.g., patients with schizophrenia and a family history of schizophrenia). Written informed consent was obtained from all subjects after the procedures were fully explained. This study was performed according to the World Medical Association’s Declaration of Helsinki and was approved by the Research Ethical Committee of Kanazawa Medical University.

### Measurement of prefrontal cortex activity using NIRS

To measure the prefrontal activity, we administered the letter version of the verbal fluency test, as described in previously published studies^34^. NIRS measurements were conducted using a 52-channel NIRS system (ETG-4000; Hitachi Medical Co., Tokyo, Japan) to detect cortical changes in the venous oxy-Hb and deoxy-Hb levels. The ETG-4000 system measures changes in the oxy-Hb and deoxy-Hb levels using 2 wavelengths (695 and 830 nm) of near-infrared light. The probe arrangement measures signal changes in the bilateral prefrontal cortex and in the superior and middle temporal cortical surface regions. Probe placement was corroborated by a multi-individual study of anatomical cranio-cerebral correction via the international 10–20 system^40^. The changes in the oxy-Hb and deoxy-Hb levels were measured using NIRS during a pre-task period (10 seconds (s), verbal fluency test-task period (60 s) and post-task period (55 s). In the verbal fluency test-task, individuals were instructed to generate as many nouns as possible that start with a Japanese *hiragana* letter (‘a’, ‘ki’, and ‘ha’, each for 20 s). During the pre- and post-task periods, individuals were also instructed to simply pronounce the syllables ‘a’, ‘i’, ‘u’, ‘e’ and ‘o’ repeatedly. The total number of correct words generated during the 60-s activation period was defined as the task performance during the NIRS measurement.

**Figure 1:**
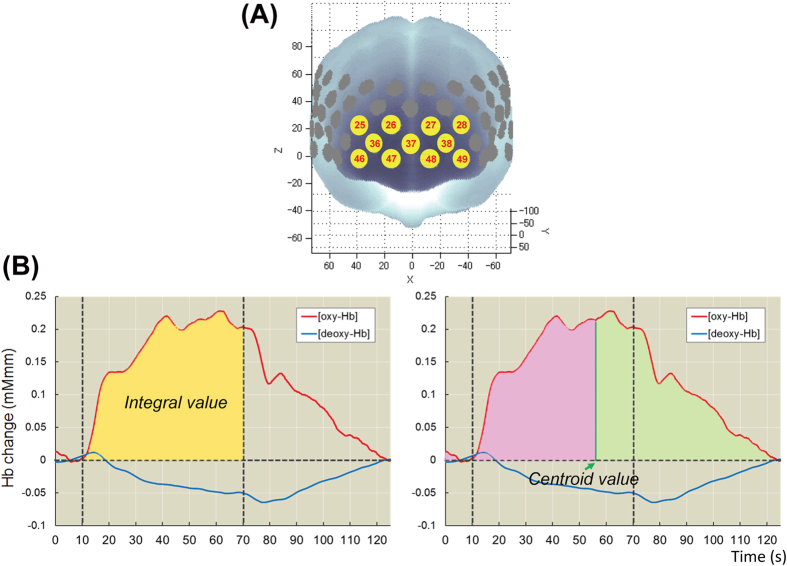
(**A**) Probe locations in the frontal region (channels 25–28, 36–38 and 46–49) using near-infrared spectroscopy (NIRS). (**B**) Depiction of the integral and centroid values as measured by NIRS.

Some studies have demonstrated acceptable reliability of the NIRS signal at the group and cluster levels rather than at the single-individual and single-channel levels^34^,^41^. On the basis of principal component analysis (PCA) of the NIRS waveforms in the previous multisite study^34^, the signals were synthesized in the clustered channels in the frontal region (11 channels, Fig. 1A). When fewer than 6 channels were available for the frontal region after software filtering, the subject data were considered unavailable for further analysis. To quantitatively extract the NIRS waveform characteristics, the integral and centroid values for the frontal region were calculated as described in a previously published study^34^. In brief, the integral value represents the brain activity intensity over time by integrating the signal changes during the verbal fluency test-task period (Fig. 1B). The centroid value represents the timing of the activity and was defined as the time (s) when the positive signal change area under the curve reached half of the total area for the full period (Fig. 1B). A low integral value reflects low brain activity in the frontal region, while a delayed centroid value reflects the inefficiency of the brain activity.

### Statistical analysis

All statistical analyses were performed using IBM SPSS Statistics 19.0 software (IBM Japan, Tokyo, Japan). Because we recruited part of a community-based sample, most demographic variables, including age and years of education, were not considered fitted to a normal distribution, as described in most clinical studies. As predicted, our demographic variables were not fitted to a normal distribution based on the Kolmogorov-Smirnov test (*p* < 5.00 × 10^−2^). Therefore, the continuous variables, such as age and years of education, were analyzed using the non-parametric Kruskal-Wallis or Mann-Whitney *U* test. The differences in categorical variables, such as gender and handedness, were analyzed using Pearson’s *χ*^*2*^ test. The effects of the diagnostic status or familial loading on prefrontal activation during the verbal fluency test were analyzed via analysis of covariance (ANCOVA) with integral or centroid values as dependent variables and diagnostic status (healthy controls, schizophrenia, major depressive disorder and bipolar disorder) or family history (healthy controls, patients with family history and patients without family history) as independent variables. Age, gender and task performance were included as covariates to control for confounding factors. *Post hoc* tests with Fisher’s least significant difference (LSD) were used to evaluate significant or marginal differences among diagnostic groups. Standardized effects were calculated using Cohen’s *d* method (http://www.uccs.edu/faculty/lbecker). The correlations between the integral or centroid values and clinical characteristics were assessed using Spearman’s non-parametric correlation coefficient. The significance level for all statistical tests was set at a two-tailed *p* < 5.00 × 10^−2^.

## Results

### Differences in prefrontal activity among healthy controls and patients with schizophrenia, major depressive disorder and bipolar disorder

**Figure 2:**
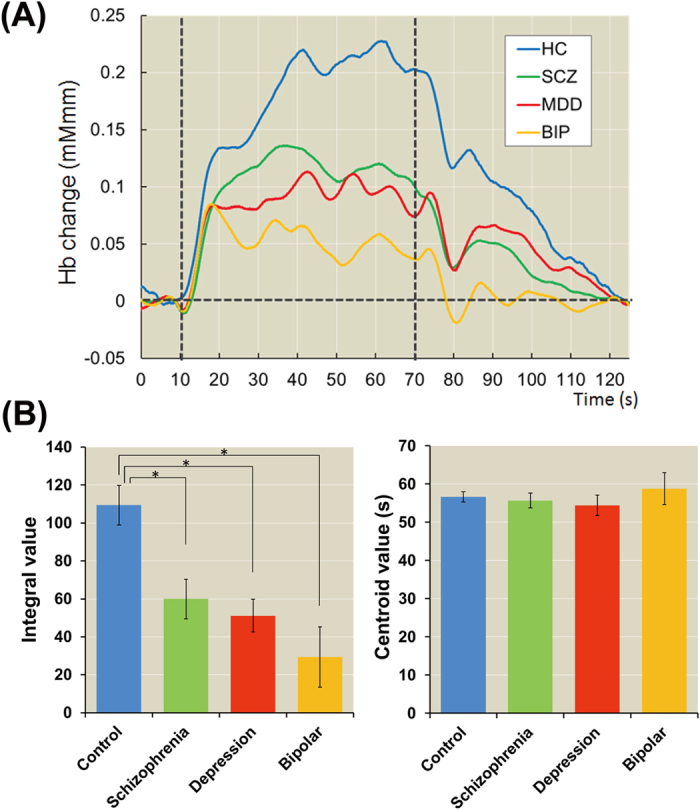
Differences in the integral and centroid values among four patient groups. (**A**) Time course of frontal region hemodynamic responses among the four diagnostic groups. (**B**) Means and standard errors of the integral and centroid values among the four groups. HC, healthy controls; SCZ, schizophrenia; MDD, major depressive disorder; and BIP, bipolar disorder. *post hoc p < 5.00 × 10^−2^.

We first investigated the diagnostic differences in integral and centroid values during the verbal fluency test among healthy individuals and patients with schizophrenia, major depressive disorder and bipolar disorder. The time courses of prefrontal hemodynamic responses among the four groups during the verbal fluency test are shown in Fig. 2A. We found significant diagnostic differences in the integral values (*F*_*3,137*_ = 8.09, *p* = 5.31 × 10^−5^) but not in the centroid values (*F*_*3,137*_ = 0.63, *p* = 6.00 × 10^−1^) among diagnostic groups (Fig. 2B). *Post hoc* analysis showed that patients with schizophrenia, major depressive disorder and bipolar disorder had lower prefrontal activity than healthy subjects (schizophrenia: Cohen’s *d* = −0.69, *p* = 5.01 × 10^−4^, depression: *d* = −0.96, *p* = 2.05 × 10^−3^, and bipolar: *d* = −1.08, *p* = 5.02 × 10^−5^). There were no differences in the integral values among patients with major psychiatric disorders (schizophrenia, major depressive disorder and bipolar disorder) or in the centroid values among the four diagnostic groups (*p* > 5.00 × 10^−2^).

### Effects of familial loading on prefrontal activity

**Figure 3:**
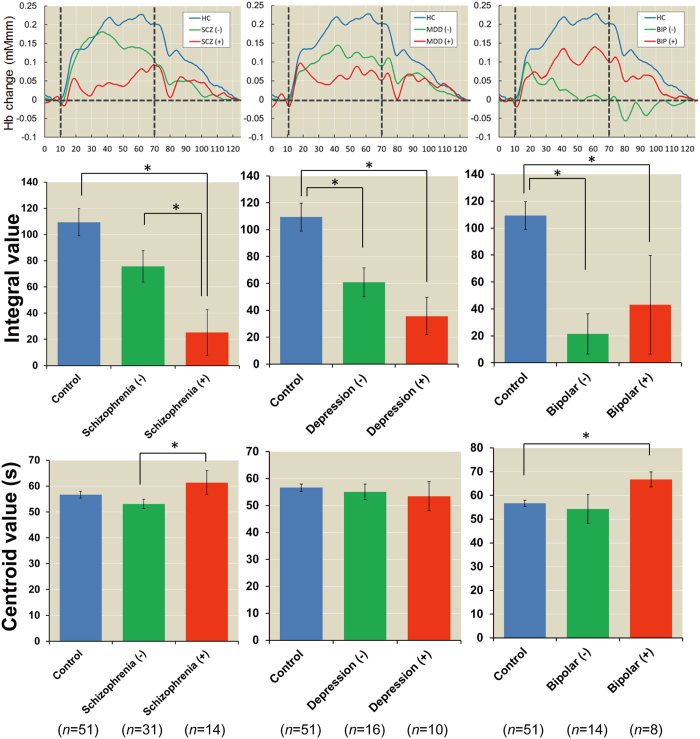
Effects of familial loading on prefrontal activity in patients with schizophrenia, major depressive disorder and bipolar disorder. (−): Negative family history of psychiatric illness and (+): Positive family history of psychiatric illness. *post hoc p < 5.00 × 10^−2^.

We next investigated the effects of a family history of psychiatric illness on prefrontal activation among healthy subjects and patients with schizophrenia, major depressive disorder or bipolar disorder with and without a family history. The demographic variables of schizophrenia, major depressive disorder and bipolar disorder patients with and without a family history are shown in Supplementary Tables 1–3, respectively. There were no differences in the demographic variables between patients with and without a family history (*p* > 5.00 × 10^−2^), except for the YMRS scores of bipolar disorder patients with and without a family history (*p* = 2.09 × 10^−2^). We found that a family history of any psychiatric illness had a significant effect on the integral values among all groups (Fig. 3, schizophrenia: *F*_*2,90*_ = 7.77, *p* = 7.69 × 10^−4^, depression: *F*_*2,71*_ = 6.15, *p* = 3.44 × 10^−3^, and bipolar: *F*_*2,67*_ = 7.19, *p* = 1.49 × 10^−3^). Patients in all diagnostic groups who had a family history [schizophrenia (+), major depressive disorder (+) and bipolar disorder (+)] had lower integral values than healthy subjects (schizophrenia: Cohen’s *d* = −1.21, *p* = 1.99 × 10^−4^, depression: *d* = −1.21, *p* = 2.14 × 10^−3^, and bipolar: *d* = −0.74, *p* = 2.89 × 10^−2^). Patients in all diagnostic groups without a family history [schizophrenia (−), major depressive disorder (−) and bipolar disorder (−)] also had lower integral values than healthy subjects (schizophrenia: *d* = −0.48, *p* = 5.35 × 10^−2^, depression: *d* = −0.80, *p* = 4.14 × 10^−2^, and bipolar: *d* = −1.34, *p* = 1.10 × 10^−3^), although the significance level in schizophrenia was marginal. Moreover, schizophrenia (+) had lower integral values than schizophrenia (−) (*d* = −0.76, *p* = 2.94 × 10^−2^). In contrast, there were no differences in the centroid values among healthy subjects and patients with or without a family history (Fig. 3, schizophrenia: *F*_*2,90*_ = 2.87, *p* = 6.22 × 10^−2^, depression: *F*_*2,71*_ = 0.15, *p* = 8.64 × 10^−1^, and bipolar: *F*_*2,67*_ = 2.27, *p* = 1.11 × 10^−1^), although schizophrenia (+) had a marginally longer centroid value than schizophrenia (−) (*d* = 0.58, *p* = 2.06 × 10^−2^) and bipolar disorder (+) had a marginally longer centroid value than bipolar disorder (−) (*d* = 0.72, *p* = 6.39 × 10^−2^) and healthy subjects (*d* = 1.09, *p* = 4.41 × 10^−2^). We examined the correlations between the integral or centroid values and clinical characteristics in each patient group with or without a family history. As shown in Supplementary Table 4, there were a few marginal correlations (*p* = 4.45 × 10^−2^ −1.53 × 10^−2^), such as between the integral value and CPZ-eq. in schizophrenia (+) patients (*ρ* = −0.63, *p* = 1.53 × 10^−2^), whereas the integral or centroid values were not significantly correlated with most of the clinical variables, such as the duration of illness, age at onset and clinical symptoms (*p* > 5.00 × 10^−2^).

To focus on family history in more detail, we investigated the effects of a family history of any major psychiatric disorder (schizophrenia, major depressive disorder or bipolar disorder) on prefrontal activation among healthy individuals and patients with or without a family history. Consistent with the effect of a family history of any psychiatric illness (Fig. 3), we found significant effects of a family history of any major psychiatric disorder on the integral value in all groups (Supplementary Fig. 1, schizophrenia: *F*_*2,90*_ = 10.25, *p* = 9.77 × 10^−5^, depression: *F*_*2,71*_ = 5.57, *p* = 5.69 × 10^−3^, and bipolar: *F*_*2,67*_ = 7.54, *p* = 1.12 × 10^−3^). Schizophrenia (+), major depressive disorder (+) and bipolar disorder (+) patients all had lower integral values than healthy subjects (schizophrenia: *d* = −1.71, *p* = 1.88 × 10^−5^, depression: *d* = −1.35, *p* = 4.95 × 10^−2^, and bipolar: *d* = −1.09, *p* = 5.77 × 10^−3^). Schizophrenia (−), major depressive disorder (−) and bipolar disorder (−) subjects also had lower integral values than healthy subjects (schizophrenia: *d* = −0.40, *p* = 9.35 × 10^−2^, depression: *d* = −0.91, *p* = 6.81 × 10^−3^, bipolar: *d* = −1.06, *p* = 3.43 × 10^−3^), although the significance level of schizophrenia was marginal. Furthermore, schizophrenia (+) patients had lower integral values than schizophrenia (−) patients (*d* = −1.25, *p* = 2.94 × 10^−3^). In contrast, there were no differences in the centroid values between healthy subjects and patients with or without a family history (Supplementary Fig. 1, schizophrenia: *F*_*2,90*_ = 2.73, *p* = 7.08 × 10^−2^, depression: *F*_*2,71*_ = 0.44, *p* = 6.44 × 10^−1^, and bipolar: *F*_*2,67*_ = 1.08, *p* = 3.47 × 10^−1^), although schizophrenia (+) patients had marginally delayed centroid values compared with schizophrenia (−) patients (*d* = 0.56, *p* = 3.39 × 10^−2^).

**Figure 4:**
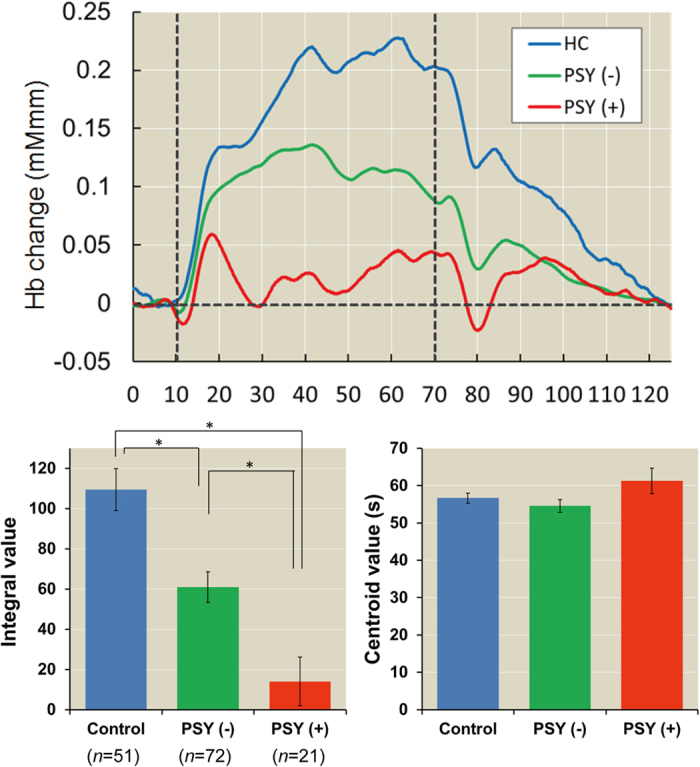
Effects of a psychiatric family history on prefrontal activity in major psychiatric disorders. When patients with schizophrenia, major depressive disorder or bipolar disorder were combined with major psychiatric disorder patients (PSY), the effects of a psychiatric family history on prefrontal activity were enhanced. PSY (−): Negative family history of any major psychiatric disorder (schizophrenia, major depressive disorder or bipolar disorder) and PSY (+): Positive family history of any major psychiatric disorder. *post hoc p < 5.00 × 10^−2^.

We added patients with schizophrenia, major depressive disorder or bipolar disorder to the group of patients with major psychiatric disorder to increase the statistical power on the basis of evidence that these disorders share common genetic factors^42^. Accordingly, the effects of a psychiatric family history on integral values were increased (Fig. 4, *F*_*2,138*_ = 17.37, *p* = 1.87 × 10^−7^). Major psychiatric disorder (+) and major psychiatric disorder (−) patients had lower integral values than healthy subjects [major psychiatric disorder (+): *d* = −1.46, *p* = 6.24 × 10^−8^ and major psychiatric disorder (−): *d* = −0.70, *p* = 3.75 × 10^−4^]. Major psychiatric disorder (+) patients had lower integral values than major psychiatric disorder (−) patients (*d* = −0.78, *p* = 1.51 × 10^−3^). We further investigated the effects of a family history of each major psychiatric disorder (schizophrenia, major depressive disorder or bipolar disorder) on prefrontal activation among healthy subjects and major psychiatric disorder patients with or without a family history. Despite the smaller sample sizes, our main findings did not change, as shown in Supplementary Figs 2 and 3.

## Discussion

This is the first study to investigate the influence of psychiatric familial loading on prefrontal activation during verbal fluency test-task performance, as measured by NIRS in patients with a major psychiatric disorder, including schizophrenia, major depressive disorder or bipolar disorder. Major psychiatric disorder patients exhibited significantly decreased prefrontal activation compared with healthy individuals. Major psychiatric patients with positive family histories (+) had significantly more severe prefrontal function impairment during the verbal fluency test compared to those with negative family histories (−). Our findings support the utility of a family history assessment in major psychiatric patients to help determine the severity of impairment.

Consistent with the results of a previously published multi-site study^34^, our study found that the integral values that represent the intensity of brain activity were significantly lower in patients with major psychiatric disorder than in healthy subjects. In contrast, the centroid values that represent activation timing were not significantly changed among all four diagnostic groups, although other studies have reported that activation timing is delayed in patients with schizophrenia and bipolar disorder compared to patients with major depressive disorder and healthy subjects^30^,^34^. We could not detect changes in the centroid values among groups because of the clinical heterogeneity of these disorders. Takizawa *et al*.^34^ have focused on patients with major psychiatric disorder who have depressive symptoms and have found differences in activation timing among major psychiatric disorders. Given that our subjects were included regardless of depressive symptoms, this difference might have affected our findings. Moreover, we found differences in activation timing when separately considering schizophrenia and bipolar disorder patients on the basis of a positive or negative family history. Schizophrenia (+) and bipolar disorder (+) patients also had delayed activation timing. These findings support the hypothesis of clinical heterogeneity in major psychiatric disorders.

The effects of psychiatric familial loading on prefrontal activation were more prominent when patients with schizophrenia, major depressive disorder and bipolar disorder were included in the group of patients with a major psychiatric disorder. Abnormal prefrontal cortical function has been found in patients with major psychiatric disorder^43^,^44^ as well as in their unaffected siblings^45^,^46^. Previously published functional imaging studies have indicated that there is a genetic contribution to prefrontal activity^47^,^48^. The cross-disorder working group of the Psychiatric Genomics Consortium (PGC) has explored shared genetic architecture across the following five main psychiatric disorders: attention deficit hyperactivity disorder, autism, bipolar disorder, major depressive disorder and schizophrenia^42^. Through analysis of disorders in 33,332 cases and 27,888 controls, the consortium has identified shared genetic variants on chromosomes 3p21 and 10q24 and within the genes for two L-type voltage-gated calcium channel subunits, *CACNA1C* and *CACNB2*. On the basis of GWAS data for schizophrenia, major depressive disorder and bipolar disorder, PGRS conferred by the additive effects of many common variants shows cross-disorder associations among adult-onset disorders (schizophrenia, major depressive disorder and bipolar disorder). This suggests that schizophrenia, major depressive disorder and bipolar disorder share genetic characteristics that do not necessarily map to specific diagnostic categories. Furthermore, the effects of the cross-disorder PGRS on prefrontal activation differ among healthy individuals with or without a family history of psychiatric illness^49^. As shown in Supplementary Table 4, clinical symptoms were not correlated with prefrontal functions, indicating that these factors are independent and that the integral and centroid values may be relatively stable over time regardless of clinical symptoms. These findings suggest that prefrontal dysfunction may be an intermediate phenotype of major psychiatric disorders and that common genetic factors might contribute to the neural mechanisms in major psychiatric disorders.

According to a previous study that sought to differentiate diagnoses at an individual level^34^, participants could optimally be classified into either the non-patient group (healthy controls; >73) or psychiatric patient group with depressive symptoms (schizophrenia, major depressive disorder and bipolar disorder; <73) when the frontal region integral value threshold was set to 73 on the basis of the receiver operating characteristic (ROC) curve. Additionally, the frontal region centroid value can differentiate between psychosis spectrum disorders (schizophrenia and bipolar disorder; >54 s) and major depressive disorder (<54 s) in patients manifesting depressive symptoms^34^. When we applied the integral and centroid value thresholds to the frontal region (Supplementary Fig. 4), 68.9%, 73.1% and 68.2% of patients with schizophrenia, major depressive disorder and bipolar disorder, respectively, were classified into patient groups using the integral value, and 51.1%, 46.2% and 72.7% of patients with schizophrenia, major depressive disorder and bipolar disorder, respectively, were classified into psychosis spectrum disorder (schizophrenia and bipolar disorder) and major depressive disorder groups by using the centroid value (Supplementary Fig. 4A). Considering the psychiatric family history of each major psychiatric disorder (schizophrenia, major depressive disorder or bipolar disorder), 100%, 100% and 50% of patients with schizophrenia (+), major depressive disorder (+) and bipolar disorder (+), respectively, were classified into the patient group by using the integral value, and 50%, 50% and 100% of patients with schizophrenia (+), major depressive disorder (+) and bipolar disorder (+), respectively, were classified into the psychosis spectrum disorder (schizophrenia and bipolar disorder) and major depressive disorder groups using the centroid values (Supplementary Fig. 4B). Although the sample size of patients with a positive family history was limited, patients with a family history also had more typical phenotypes. Further research using larger sample sizes of patients with a positive family history may be warranted.

There are several limitations to this study. First, the sample sizes of the subjects in the compared groups were quite distinct for all comparison data analyses. If the sample sizes are equal or close among the groups, comparison is fairer and makes the results/findings more convincing. In contrast, if the sample sizes are decreased for the comparison, there would statistically be more false positive or false negative findings. Therefore, it would be better to increase the sample sizes. The psychiatric family history in this study was derived from information obtained from patients and their families through clinical interviews and/or checklists. In this way, we did not directly diagnose relatives. Our participants consisted of a community-based sample of patients with schizophrenia, major depressive disorder, bipolar disorder and healthy subjects. Therefore, demographic variables such as age and gender may not have been matched among diagnostic groups, although these variables were treated as covariates. We could not completely exclude the influences of medications on prefrontal activation or the duration of illness, although previous studies have reported no association between medications and prefrontal activity^34^,^50^. However, the degree of prefrontal activation decreased in accordance with the clinical stage of psychosis^27^. Our results might have been affected by these factors. A wide range of prefrontal functions are impaired in patients with major psychiatric disorder^51^. The present study only examined prefrontal function during the verbal fluency task. As there are prefrontal dysfunctions that we did not examine, such as working memory, that are also observed in major psychiatric disorders, further studies are needed to investigate the effect of a family history of major psychiatric disorders at different function levels.

In conclusion, we found that psychiatric familial loading affects prefrontal activation in major psychiatric disorder patients during the verbal fluency test, as measured by NIRS. Compared with patients who lacked a psychiatric family history, patients with a family history had more severe prefrontal dysfunction. Our results provide further support for the investigation of genetic factors underlying both major psychiatric disorders and prefrontal activation.

## Additional Information

**How to cite this article:** Ohi, K. *et al*. Impact of Familial Loading on Prefrontal Activation in Major Psychiatric Disorders: A Near-Infrared Spectroscopy (NIRS) Study. *Sci. Rep.* **7**, 44268; doi: 10.1038/srep44268 (2017).

**Publisher's note:** Springer Nature remains neutral with regard to jurisdictional claims in published maps and institutional affiliations.

## Supplementary Material

Supplementary Information

## Figures and Tables

**Table 1:** Demographic variables of the included diagnostic groups.

Variables	SCZ	MDD	BIP	HC	*p* values (*χ*^*2*^)
	(*n* = 45)	(*n* = 26)	(*n* = 22)	(*n* = 51)	
Age (years)	35.4 ± 9.1	41.1 ± 12.7	39.9 ± 12.5	35.7 ± 11.9	6.45 × 10^−2^ (7.3)
Gender (male/female)	16/29	17/9	13/9	33/18	**1.78 ** × ** 10** ^**−2**^ **(10.1)** ^a^
Education (years)	13.2 ± 1.3	14.3 ± 2.6	14.7 ± 2.3	16.7 ± 2.5	**2.61 ** × ** 10** ^**−10**^ **(47.6)**
Estimated premorbid IQ	101.3 ± 11.6	104.6 ± 9.2	110.2 ± 6.2	110.0 ± 6.2	**1.82 ** × ** 10** ^**−4**^ **(19.9)**
Handedness (rt./lt./bil.)	40/3/2	24/2/0	19/1/2	46/4/1	7.20 × 10^−1^ (3.7)^a^
Performance	13.0 ± 5.4	15.7 ± 5.3	16.9 ± 5.3	15.3 ± 4.7	**4.18 ** × ** 10** ^**−2**^ **(8.2)**
CPZeq. (mg/day)	465.3 ± 407.3	51.4 ± 88.6	76.7 ± 131.8	—	**1.45 ** × ** 10** ^**−11**^ **(49.9)**
Age at onset (years)	25.5 ± 6.4	33.8 ± 13.5	30.6 ± 9.3	—	**4.05 ** × ** 10** ^**−3**^ **(11.0)**
Duration of illness (years)	9.8 ± 8.7	7.3 ± 6.4	9.3 ± 9.7	—	6.32 × 10^−1^ (0.9)
Family history^b^					
SCZ	8	0	1	0	—
MDD	7	2	3	0	—
BIP	0	1	3	0	—
Other diagnosis	2	7	3	0	—
Unknown	0	3	1	0	—

SCZ, schizophrenia; MDD, major depressive disorder; BIP, bipolar disorder; HC, healthy controls; IQ, intelligence quotient; and CPZeq; chlorpromazine equivalents of total antipsychotics. Means ± SD are shown. Complete demographic information was not obtained for all subjects (estimated premorbid IQ in SCZ, *n* = 43; in MDD, *n* = 23). ^a^*χ*^*2*^ test. ^b^The cumulative number is indicated. Significant *p* values are shown in boldface and underlined.
