# Supplementary material for: Impact of Familial Loading on Prefrontal Activation in Major Psychiatric Disorders: A Near-Infrared Spectroscopy (NIRS) Study
Source: Sci Rep. 2017 Mar 15;7:44268. doi: 10.1038/srep44268 (PMC5353718; doi:10.1038/srep44268)
Supplement: Supplementary Information [file srep44268-s1.doc]

**Impact of Familial Loading on Prefrontal Activation in Major Psychiatric Disorders:**

**A Near-Infrared Spectroscopy (NIRS) Study**

Kazutaka Ohi^1^,*, Takamitsu Shimada^1^, Hiroaki Kihara^1^, Toshiki Yasuyama^1^, Kazuyuki Sawai^1^, Yukihisa Matsuda^1, 2^, Kazuaki Oshima^1^, Hiroaki Okubo^1^, Yusuke Nitta^1^, Takashi Uehara^1^ & Yasuhiro Kawasaki^1^

^1^ Department of Neuropsychiatry, Kanazawa Medical University, 1-1 Daigaku, Uchinada, Ishikawa, 920-0293, Japan.

^2^ Project Research Center, Kanazawa Medical University, 1-1 Daigaku, Uchinada, Ishikawa, 920-0293, Japan.

* Correspondence and requests for materials should be addressed to K.O. (email: [ohi@kanazawa-med.ac.jp](mailto:ohi@kanazawa-med.ac.jp))

**
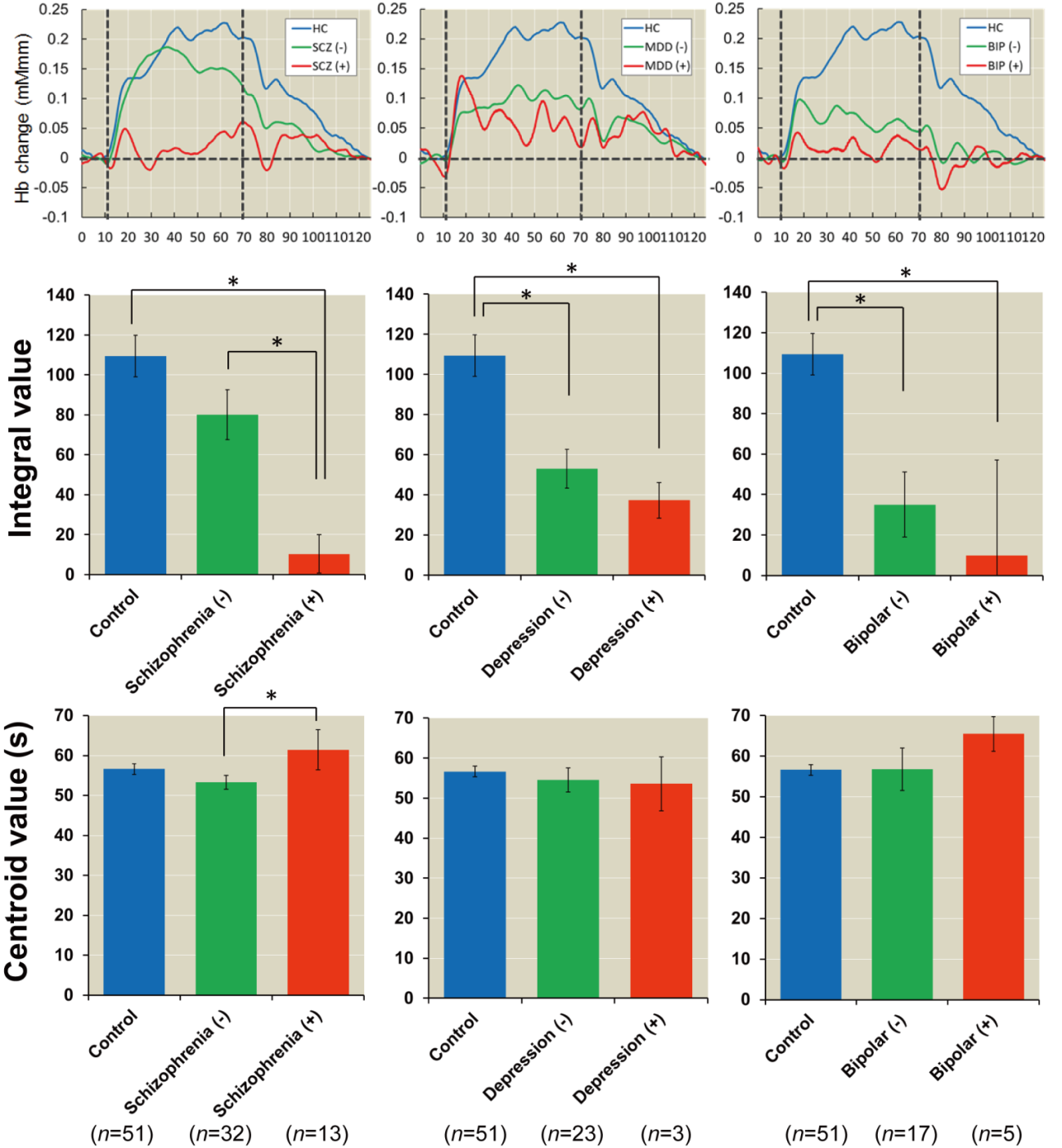
**

**Supplementary Fig. 1** Effects of familial loading on prefrontal activation in patients with schizophrenia, major depressive disorder and bipolar disorder. (-): Negative family history of any major psychiatric disorder (schizophrenia, major depressive disorder or bipolar disorder) and (+): Positive family history of any major psychiatric disorder. **post hoc* *p*<5.00×10^-2^**.**

**
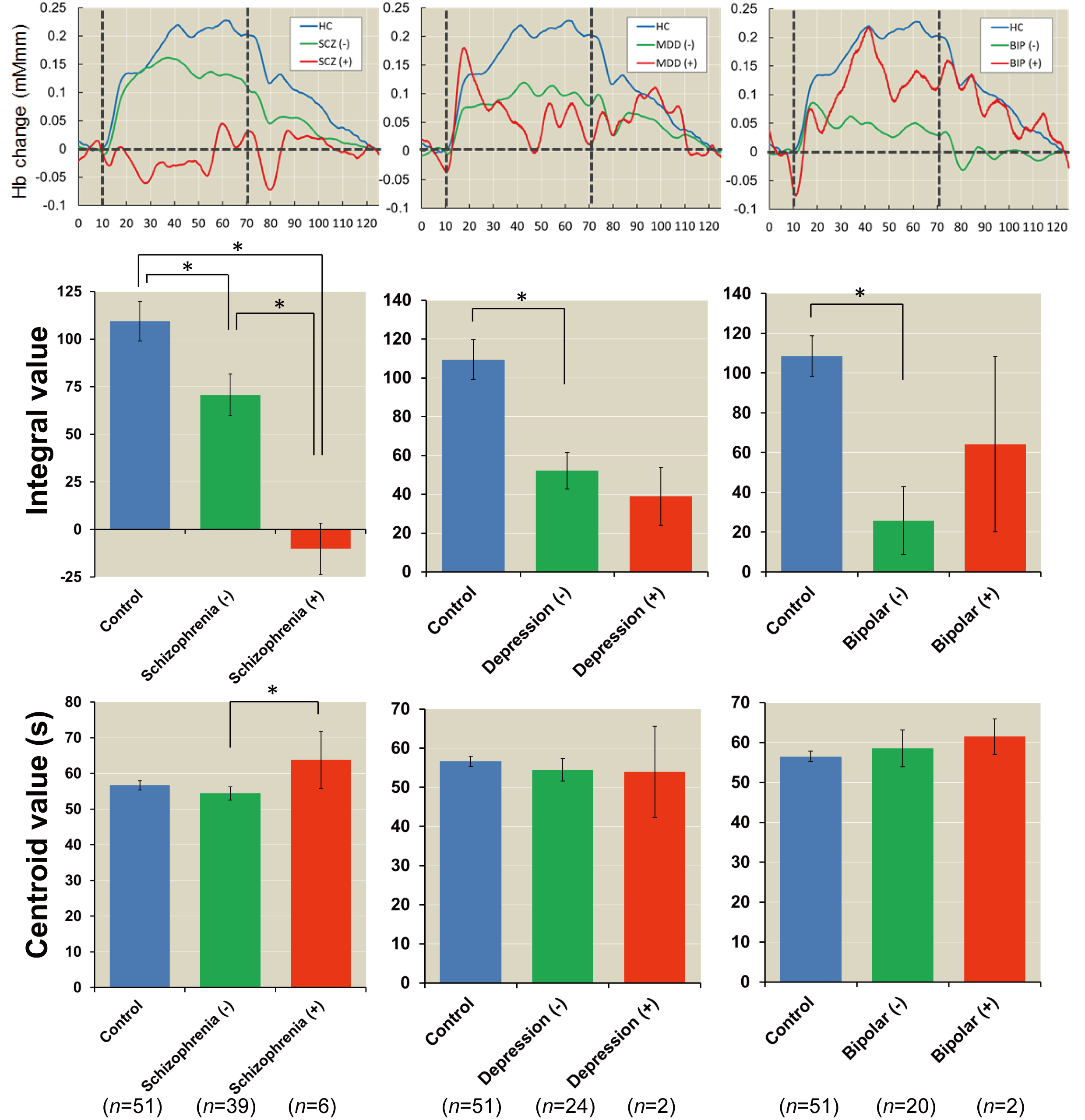
**

**Supplementary Fig. 2** Effects of familial loading on prefrontal activation in patients with schizophrenia, major depressive disorder and bipolar disorder. (-): Negative family history of each major psychiatric disorder (schizophrenia, major depressive disorder or bipolar disorder) and (+): Positive family history of each major psychiatric disorder (schizophrenia, major depressive disorder or bipolar disorder: e.g., patients with schizophrenia and a family history of schizophrenia). **post hoc* *p*<5.00×10^-2^**.**

**
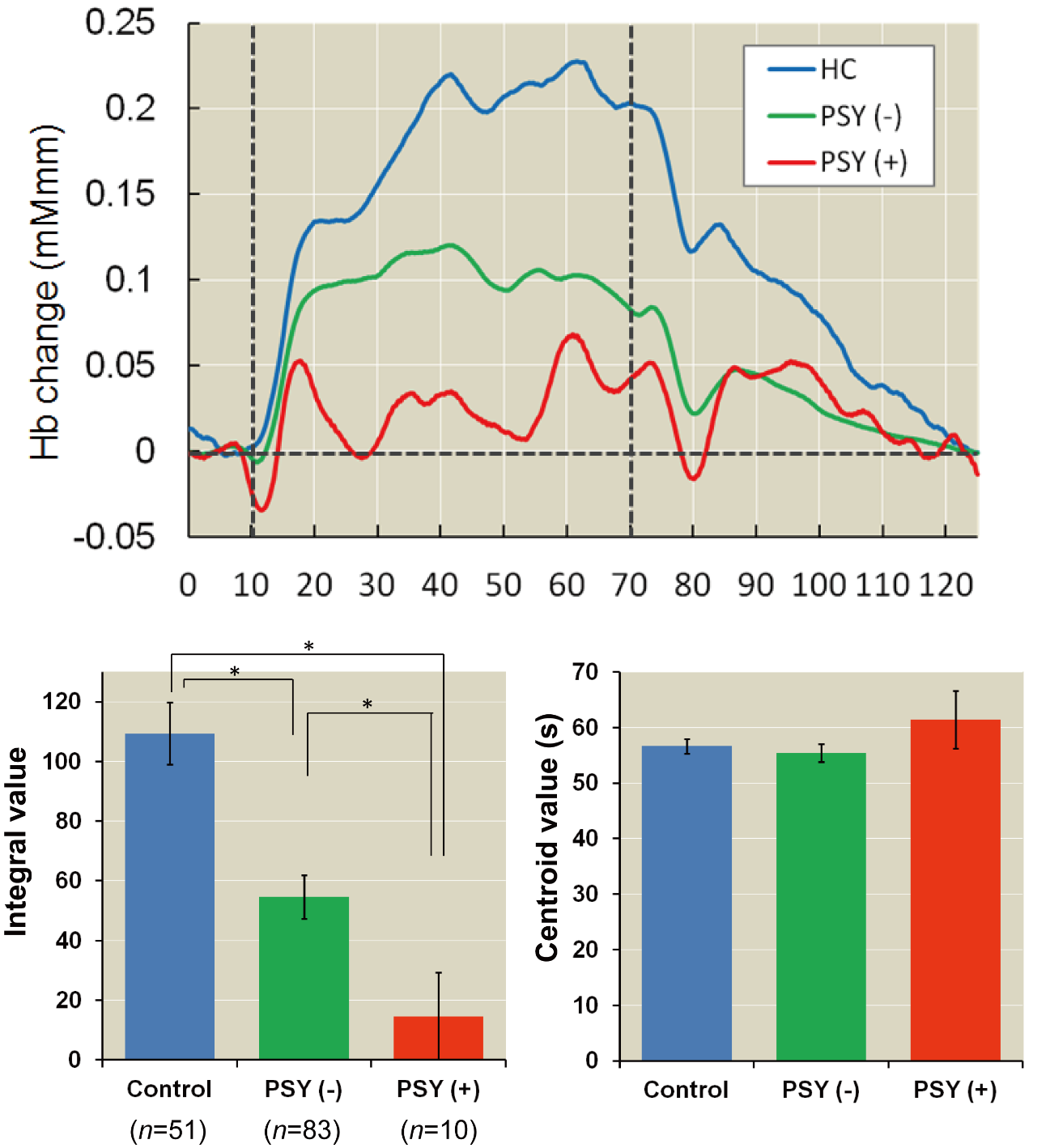
**

**Supplementary Fig. 3** Effects of a psychiatric family history on prefrontal activity. PSY (-): Negative family history of each major psychiatric disorder (schizophrenia, major depressive disorder or bipolar disorder) and PSY (+): Positive family history of each major psychiatric disorder. **post hoc* *p*<5.00×10^-2^**.**

**
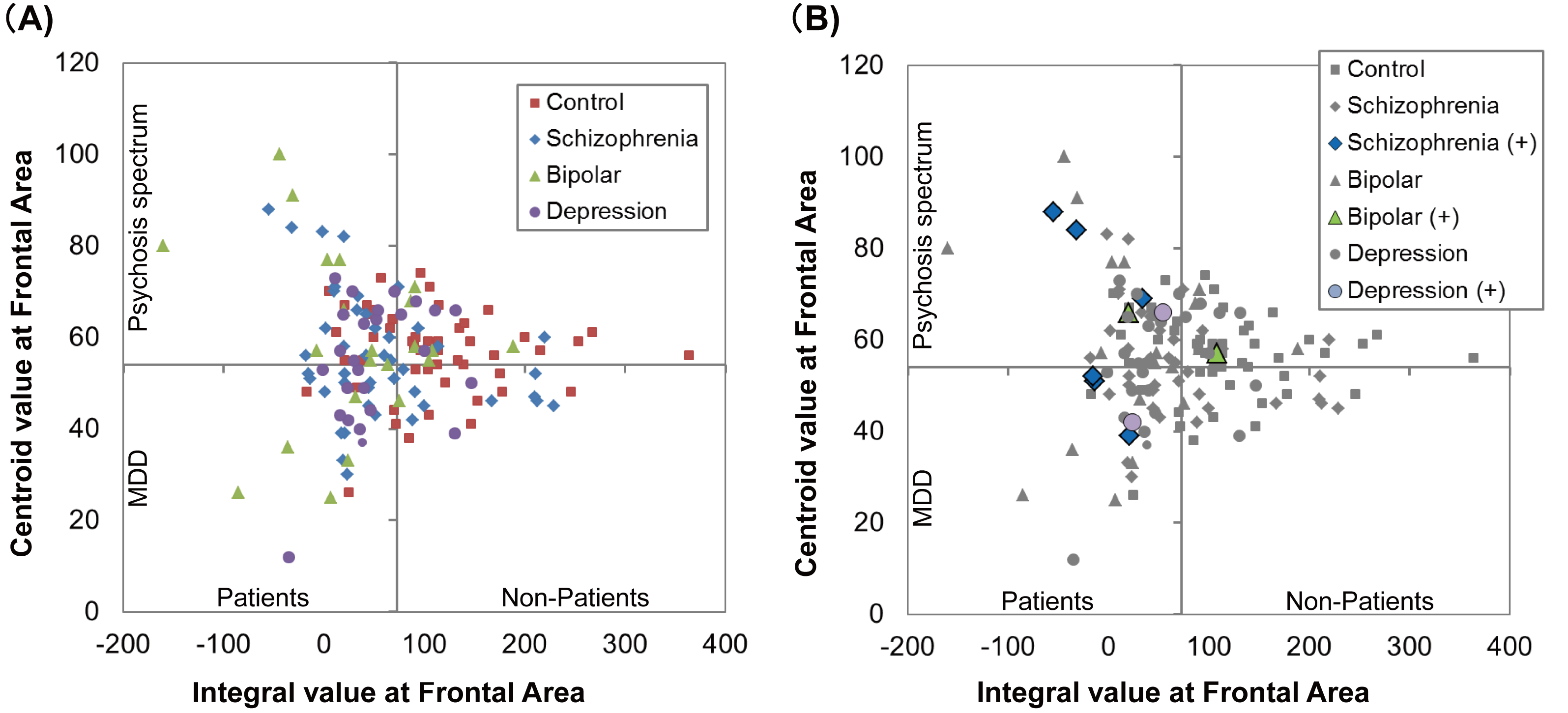
**

**Supplementary Fig. 4** Scatter plots of the integral and centroid values. When the frontal region integral value threshold was set to 73 on the basis of a previous ROC curve (Takizawa et al., 2014), participants could be optimally classified into either the non-patient group (HC; >73) or psychiatric patient group with depressive symptoms (schizophrenia, major depressive disorder and bipolar disorder; <73). Additionally, the frontal region centroid value could differentiate between psychosis spectrum disorders (schizophrenia and bipolar disorder; >54 s) and major depressive disorder (<54 s) in patients manifesting depressive symptoms. When we applied the integral and centroid value thresholds to the frontal region, 68.9%, 73.1% and 68.2% of patients with schizophrenia, major depressive disorder and bipolar disorder, respectively, were classified into the patient group using the integral values, and 51.1%, 46.2% and 72.7% of patients with schizophrenia, major depressive disorder and bipolar disorder, respectively, were classified into the psychosis spectrum disorders (schizophrenia and bipolar disorder) and major depressive disorder groups using the centroid value **(A)**. Considering the psychiatric family history of each major psychiatric disorder (schizophrenia, major depressive disorder or bipolar disorder), 100%, 100% and 50% of patients with schizophrenia (+), major depressive disorder (+) and bipolar disorder (+), respectively, were classified into the patient group using the integral values, and 50%, 50% and 100% of patients with schizophrenia (+), major depressive disorder (+) and bipolar disorder (+), respectively, were classified into the psychosis spectrum disorders (schizophrenia and bipolar disorder) and major depressive disorder groups using the centroid values **(B)**.

**Supplementary Table 1 Demographic variables for the schizophrenia patient groups**

|  | Schizophrenia | |  |
| --- | --- | --- | --- |
|  | FH (-) | FH (+) |  |
| Variables | (*n*=31) | (*n*=14) | *p* values (*z*) |
| Age (years) | 35.2 ± 9.0 | 35.9 ± 9.7 | 8.64×10^-1^ (0.17) |
| Gender (male/female) | 10/21 | 6/8 | 4.92×10^-1^ (0.47)^a^ |
| Education (years) | 13.1 ± 1.3 | 13.2 ± 1.4 | 9.79×10^-1^ (0.03) |
| Estimated premorbid IQ | 103.0 ± 11.9 | 97.4 ± 10.5 | 1.28×10^-1^ (-1.52) |
| Handedness (rt./lt./bil.) | 27/3/1 | 13/0/1 | 4.22×10^-1^ (1.72)^a^ |
| Performance | 12.8 ± 5.4 | 13.4 ± 5.8 | 8.54×10^-1^ (0.18) |
| CPZeq. (mg/day) | 486.7 ± 464.6 | 418.0 ± 245.0 | 6.67×10^-1^ (0.43) |
| Age at onset (years) | 24.9 ± 5.9 | 26.9 ± 7.4 | 4.10×10^-1^ (0.82) |
| Duration of illness (years) | 10.3 ± 8.9 | 8.9 ± 8.5 | 8.34×10^-1^ (-0.21) |
| PANSS positive symptoms | 14.3 ± 5.3 | 14.2 ± 5.6 | 7.75×10^-1^ (-0.29) |
| PANSS negative symptoms | 16.2 ± 6.5 | 18.0 ± 7.4 | 4.43×10^-1^ (0.77) |
| PANSS general psychopathology | 30.6 ± 7.1 | 33.9 ± 10.5 | 2.99×10^-1^ (1.04) |

FH, family history; IQ, intelligence quotient; and CPZeq; chlorpromazine equivalents of total antipsychotics. Means ± SD are shown. Complete demographic information was not obtained for all subjects (estimated premorbid IQ in schizophrenia (-), *n*=30; in schizophrenia (+), *n*=13; PANSS in schizophrenia (-), *n*=29). ^a^*χ^2^* test. Other group differences in the demographic variables were compared using the Mann-Whitney *U* test.

**Supplementary Table 2 Demographic variables for the major depressive disorder patient groups**

|  | Major depressive disorder | |  |
| --- | --- | --- | --- |
|  | FH (-) | FH (+) |  |
| Variables | (*n*=16) | (*n*=10) | *p* values (*z*) |
| Age (years) | 42.1 ± 13.7 | 39.5 ± 11.4 | 8.95×10^-1^ (-0.13) |
| Gender (male/female) | 9/7 | 8/2 | 2.16×10^-1^ (1.53)^a^ |
| Education (years) | 14.3 ± 2.9 | 14.4 ± 2.1 | 7.71×10^-1^ (0.29) |
| Estimated premorbid IQ | 104.3 ± 9.5 | 104.9 ± 9.1 | 8.50×10^-1^ (0.19) |
| Handedness (rt./lt./bil.) | 14/2/0 | 10/0/0 | 2.45×10^-1^ (1.35)^a^ |
| Performance | 14.7 ± 4.6 | 17.3 ± 6.3 | 3.28×10^-1^ (0.98) |
| CPZeq. (mg/day) | 44.5 ± 79.7 | 62.5 ± 104.9 | 6.19×10^-1^ (0.50) |
| Age at onset (years) | 35.9 ± 14.5 | 30.5 ± 11.6 | 5.61×10^-1^ (-0.58) |
| Duration of illness (years) | 6.3 ± 6.7 | 9.0 ± 5.9 | 1.75×10^-1^ (1.36) |
| HAMD-17 | 13.4 ± 6.1 | 12.6 ± 5.2 | 8.15×10^-1^ (-0.23) |

Complete demographic information was not obtained for all subjects, such as the estimated premorbid IQ in major depressive disorder (-), *n*=14; in major depressive disorder (+), *n*=9; HAMD-17 in major depressive disorder (-), *n*=13; in major depressive disorder (+), *n*=9). ^a^*χ^2^* test. Other group differences in the demographic variables were compared using the Mann-Whitney *U* test.

**Supplementary Table 3 Demographic variables for the bipolar disorder patient groups**

|  | Bipolar disorder | |  |
| --- | --- | --- | --- |
|  | FH (-) | FH (+) |  |
| Variables | (*n*=14) | (*n*=8) | *p* values (*z*) |
| Age (years) | 40.9 ± 12.5 | 38.1 ± 13.1 | 4.94×10^-1^ (-0.68) |
| Gender (male/female) | 7/7 | 6/2 | 2.51×10^-1^ (1.32)^a^ |
| Education (years) | 15.1 ± 2.4 | 14.0 ± 1.9 | 3.13×10^-1^ (-1.01) |
| Estimated premorbid IQ | 110.7 ± 6.5 | 109.3 ± 6.1 | 7.84×10^-1^ (-0.27) |
| Handedness (rt./lt./bil.) | 13/0/1 | 6/1/1 | 3.50×10^-1^ (2.10)^a^ |
| Performance | 17.2 ± 4.7 | 16.4 ± 6.4 | 5.37×10^-1^ (-0.62) |
| CPZeq. (mg/day) | 85.7 ± 159.5 | 60.9 ± 66.3 | 7.47×10^-1^ (0.32) |
| Age at onset (years) | 30.9 ± 8.6 | 30.0 ± 11.0 | 5.16×10^-1^ (-0.65) |
| Duration of illness (years) | 10.0 ± 9.7 | 8.1 ± 10.4 | 4.50×10^-1^ (-0.76) |
| YMRS | 5.7 ± 4.8 | 1.0 ± 2.8 | **2.09×10^-2^ (-2.31)** |

Complete demographic information was not obtained for all subjects (YMRS in bipolar disorder (-), *n*=13). ^a^*χ^2^* test. Other group differences in the demographic variables were compared using the Mann-Whitney *U* test. Significant *p* values are shown in boldface and underlined.

**Supplementary Table 4 Correlations between the integral or centroid values and clinical characteristics in each patient group with or without a family history**

|  | Schizophrenia | | Depression | | Bipolar | |
| --- | --- | --- | --- | --- | --- | --- |
|  | FH (-) | FH (+) | FH (-) | FH (+) | FH (-) | FH (+) |
| ***Integral value*** |  |  |  |  |  |  |
| Age | -0.15 | -0.33 | -0.39 | 0.26 | 0.18 | -0.17 |
| Gender (M=1, F=2) | -0.13 | 0.11 | 0.15 | 0.17 | 0.44 | 0.13 |
| Education | -0.06 | -0.26 | 0.18 | 0.14 | -0.04 | -0.38 |
| Estimated premorbid IQ | 0.05 | -0.04 | 0.19 | -0.01 | -0.13 | -0.22 |
| Performance | -0.22 | -0.13 | 0.48 | -0.31 | -0.06 | -0.53 |
| CPZeq. | -0.02 | -0.63* | 0.39 | 0.14 | 0.24 | 0.62 |
| Age at onset | 0.18 | 0.10 | -0.32 | -0.24 | -0.02 | 0.13 |
| Duration of illness | -0.28 | -0.52 | 0.01 | 0.61 | 0.25 | -0.49 |
| PANSS positive symptoms | -0.17 | 0.15 | - | - | - | - |
| PANSS negative symptoms | -0.09 | -0.08 | - | - | - | - |
| PANSS general psychopathology | -0.18 | 0.02 | - | - | - | - |
| HAMD-17 | - | - | -0.02 | -0.36 | - | - |
| YMRS | - | - | - | - | -0.31 | 0.41 |
| ***Centroid value*** |  |  |  |  |  |  |
| Age | -0.11 | -0.42 | 0.05 | 0.20 | 0.02 | 0.43 |
| Gender (M=1, F=2) | -0.16 | -0.21 | 0.53* | -0.09 | -0.37 | 0.13 |
| Education | 0.04 | -0.07 | 0.35 | 0.14 | 0.19 | 0.69 |
| Estimated premorbid IQ | -0.06 | 0.10 | 0.15 | -0.31 | -0.06 | 0.57 |
| Performance | -0.10 | 0.24 | 0.22 | -0.65* | -0.26 | 0.02 |
| CPZeq. | 0.31 | 0.37 | 0.01 | 0.06 | -0.16 | -0.25 |
| Age at onset | 0.04 | -0.33 | -0.03 | -0.02 | -0.20 | -0.07 |
| Duration of illness | -0.03 | -0.05 | 0.41 | 0.22 | 0.15 | 0.63 |
| PANSS positive symptoms | 0.01 | -0.09 | - | - | - | - |
| PANSS negative symptoms | 0.08 | -0.08 | - | - | - | - |
| PANSS general psychopathology | 0.19 | 0.12 | - | - | - | - |
| HAMD-17 | - | - | 0.11 | -0.36 | - | - |
| YMRS | - | - | - | - | -0.56* | -0.41 |

FH, family history; M, male; F, female; PANSS, the Positive and Negative Syndrome Scale; HAMD-17, the 17-item Hamilton Rating Scale for Depression; and YMRS, the Young Mania Rating Scale. Spearman’s *ρ* scores are shown. **p*<5.00×10^-2^.
